# Biomarker Identification through Proteomics in Colorectal Cancer

**DOI:** 10.3390/ijms25042283

**Published:** 2024-02-14

**Authors:** Desirée Martín-García, Marilina García-Aranda, Maximino Redondo

**Affiliations:** 1 Surgical Specialties, Biochemistry and Immunology Department, Faculty of Medicine, University of Málaga, 29010 Málaga, Spain; desirermg@uma.es; 2 Red de Investigación en Cronicidad, Atención Primaria y Promoción de la Salud (RICAPPS), 29590 Málaga, Spain; marilina.garcia.sspa@juntadeandalucia.es; 3 Instituto de Investigación Biomédica de Málaga y Plataforma en Nanomedicina—IBIMA Plataforma BIONAND, 29590 Málaga, Spain; 4 Research and Innovation Unit, Hospital Universitario Costa del Sol, 29602 Marbella, Spain

**Keywords:** colorectal cancer, proteomics, biomarkers, personalized medicine, targeted treatment

## Abstract

Colorectal cancer (CRC) is a devastating disease that ranks third in diagnosis and as the second leading cause of cancer-related deaths. The early detection of CRC has been shown to be the most effective strategy to improve treatment outcomes and patient survival. Therefore, current lines of research focus on the development of reliable diagnostic tools. Targeted therapies, in combination with standard chemotherapy and immune checkpoint inhibitors, have emerged as promising treatment protocols in CRC. However, their effectiveness is linked to the molecular characteristics of each patient. The importance of discovering biomarkers that help predict response to therapies and assess prognosis is evident as they allow for a fundamental step towards personalized care and successful treatments. Among the ongoing efforts to identify them, mass spectrometry-based translational proteomics presents itself as a unique opportunity as it enables the discovery and application of protein biomarkers that may revolutionize the early detection and treatment of CRC. Our objective is to show the most recent studies focused on the identification of CRC-related protein markers, as well as to provide an updated view of advances in the field of proteomics and cancer.

## 1. Introduction

A biomarker constitutes a measurable indicator of a specific biological state, especially related to the risk of developing a disease, its presence, or its stage of development. While in the past, the concept commonly referred to physical traits or physiological metrics, nowadays, the term is more frequently used to describe its molecular nature. Molecular biomarkers can manifest in various ways, leading to the implementation of diverse strategies for their discovery.

Although transcriptomic and DNA methylation profiling studies have proven highly effective in discovering biomarkers in the context of cancer [1], information derived from DNA or RNA alone is not entirely suitable for determining the best cancer drugs. This is because most drugs against this disease target specific proteins. For this reason, metabolomic approaches are showing promising results in the study of metabolic diseases, drugs, and associated toxicity [2]. In this regard, it is not always straightforward to relate a genetic mutation to the expected change in the corresponding protein. With this pretext, proteomics has positioned itself in recent years as a particularly promising tool for biomarker discovery.

Biomarkers can have fundamental clinical applications, including the detection, diagnosis, or monitoring of disease activity, as well as guiding molecularly targeted therapies or evaluating therapeutic responses. In the biopharmaceutical industry, biomarkers define molecular classifications of patients and diseases and act as surrogate criteria in the early phases of clinical trials for drugs.

**Figure 1:**
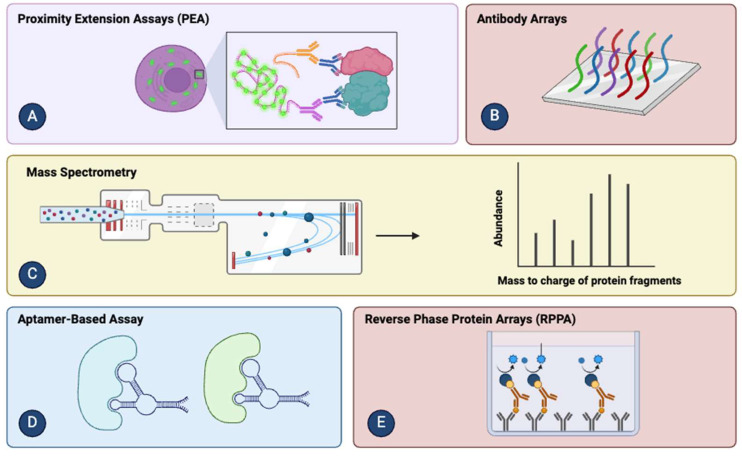
High-Complexity Proteomic Technologies: (**A**) proximity detection assays are based on the binding of the analyte through two proximity probes to the target [6]; (**B**) antibody–antigen arrays—antibodies are printed on a solid support, and, subsequently, the sample is applied to them where the antigen–antibody interaction can be achieved through various methods [5]; (**C**) mass spectrometry combined with other techniques is used to retrieve peptide masses and high-precision fragment spectra from digestion products specific to protein sequence [3]; (**D**) aptamer-based detection assays rely on their ability to bind to almost any protein specifically [7]; (**E**) reverse-phase protein arrays combine microdissection of histologically relevant cell populations with probing using antibodies that can be detected through fluorescent, colorimetric, or chemiluminescent assays [4]. Images were created using Biorender.com (accessed on 12 February 2024).

The current demand for new proteomic biomarkers has sparked a special interest in developing new technologies to understand the proteome. Currently, high-complexity proteomic technologies, both conventional and innovative, include mass spectrometry [3], reverse-phase protein arrays [4], antibody/antigen/bead arrays [5], proximity extension assays [6], and aptamer assays [7] (Figure 1).

Each of them has an analytical scope to characterize hundreds to thousands of protein targets simultaneously from a single sample, along with a set of advantages and disadvantages outlined in Table 1.

In the last ten years, there has been an exponential increase in the number of scientific articles published related to the identification of biomarkers using each of these techniques, especially through mass spectrometry, a technique for which up to 1500 publications per year are reported, accumulating up to 21,800 since 2004. Given the importance of staying informed about advances in the field of molecular analyses and the potential identification of biomarkers, a literature review was conducted on possible specific biomarkers for CRC. Articles published between 2000 and 2023 were sought with the keyword “biomarkers in CRC” in databases that store original scientific articles, such as PubMed or Scopus databases. The last search was performed on 9 February 2024.

## 2. Molecular Complexity of Colorectal Cancer

Colorectal cancer (CRC) ranks third in incidence, accounting for approximately 10% of all cases worldwide, trailing only breast cancer and lung cancer [8]. Overall, its incidence varies by geographical region and population risk factors, being more prevalent in developed countries, although its incidence is rising in developing countries [8]. Risk factors can be lifestyle and health-related, such as alcohol consumption, smoking, a diet high in fats and low in vegetables, obesity, and lack of physical activity, or intrinsic and non-modifiable factors, such as age, ethnicity, and genetic predisposition [9]. It is estimated that between 15% and 30% of CRC cases have a hereditary component, occurring more frequently in individuals with first- and second-degree affected relatives [10]. Inflammatory bowel diseases, such as Crohn’s disease and ulcerative colitis, also increase the risk of CRC, especially when inflammation is chronic and long-lasting [11].

**Figure 2:**
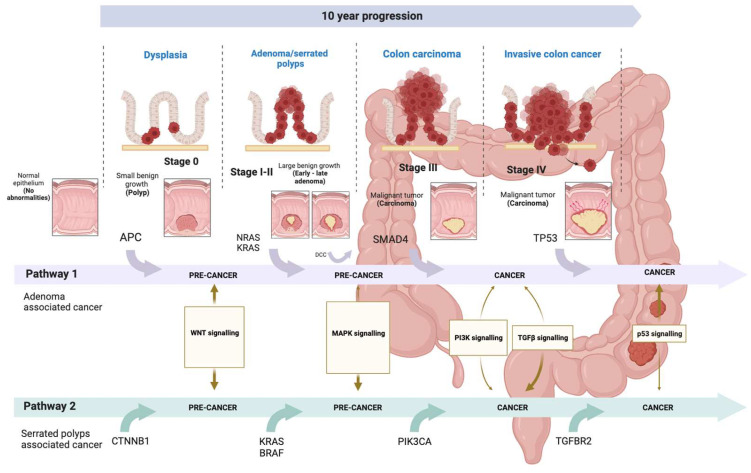
Evolution of CRC. The evolution of CRC involves two pathways. Firstly, the adenoma-carcinoma pathway, characterized by the accumulation of mutations in the WNT, MAPK, PI3K, TGFβ, and p53 signaling pathways, leading to a sequential progression from adenoma to carcinoma. On the other hand, there is the serrated pathway, featuring specific mutations in the KRAS or BRAF genes, resulting in hyperactivation of the MAPK signaling pathway [21,24]. The stages of CRC are determined by the extent of the tumor and the presence of metastases. The TNM staging system assesses cancer, focusing on Tumor (T), which describes the depth of primary tumor growth into the intestinal lining (ranging from T0 to T4b, indicating invasion of other organs or structures). The evaluation of Lymph Nodes (N) and Metastases (M) is combined with T to assign a stage to the cancer, ranging from 0 to IV [12]. Images were created using Biorender.com (accessed on 12 February 2024).

CRC is classified into different stages based on the extent of the tumor and the presence of metastasis (Figure 2) [12]. In stage 0 (carcinoma in situ), cancer cells are confined to the innermost layer of the lining of the colon or rectum, without invading nearby tissues or spreading to lymph nodes or other parts of the body. In stage I, the cancer grows beyond the inner lining but is not spread to lymph nodes or distant organs. In stage II, it grows through the lining but does not reach lymph nodes or distant organs. In stage III, it invades nearby lymph nodes but does not reach distant organs. In stage IV, cancer spreads to distant organs. The prognosis and treatment vary at each stage and may include surgery, radiotherapy, chemotherapy, and targeted or immunotherapy.

In terms of mortality, CRC holds the second position, with a survival rate of approximately 65% [13,14]. Unfortunately, approximately 25% of patients have been observed to delay seeking medical attention [15,16], resulting in 60% of patients being in an advanced stage of the disease, and an alarming 22% presenting distant metastasis at the time of diagnosis in 2019 [17]. This leads to lower survival rates, as they have been found to be significantly better when detected early, with a 91% rate at 5 years in stage I, decreasing to 72% in advanced stages and dropping dramatically to 14% in stage IV [17].

CRC involves various pathophysiological mechanisms, such as cell differentiation, abnormal cell proliferation, resistance to apoptosis, invasion of adjacent structures, and distant metastasis (Figure 2) [18,19], as well as molecular alterations involving certain genes and the interaction of multiple signaling pathways with a complex mechanism that is not yet well understood [20]. Understanding these pathways is crucial for the development of targeted therapies and more effective treatment strategies.

A significant portion of CRC cases is sporadic and develops slowly over several years following an adenoma–carcinoma sequence, perfectly described by what is known as the Vogelstein model [21]. In this model, mutations accumulate in the WNT, MAPK, TGFβ, and p53 signaling pathways, marking the initiation and progression of CRC sequentially. Mutations in the *APC* (Adenomatous Polyposis Coli) gene occur in 70% of colorectal adenoma cases, which progress to carcinoma by acquiring activating mutations in *KRAS* and inactivating mutations in *SMAD4* and *TP53* (Tumor Protein 53). The hyperactivation of the WNT signaling pathway usually arises from mutations in the *APC* gene. *APC* is a negative regulator of the WNT pathway, part of the Axin–APC complex promoting the proteasomal degradation of B-catenin, a WNT effector. If the complex is defective due to *APC* inactivation, excess B-catenin accumulates in the cytoplasm and translocates to the nucleus, where it activates *MYC* and other genes. This disruption leads to the dysregulation of cell proliferation and differentiation, favoring the development of dysplastic crypts and the progression of adenomas to carcinomas, usually associated with mutations in the tumor suppressor gene *TP53* [22,23].

In addition to the described adenoma–carcinoma sequence, an estimated 10% to 20% of colorectal carcinomas develop through a different pathway known as the serrated pathway. While most serrated polyps are typically considered benign lesions, a subset of serrated lesions can progress to carcinoma. A distinctive feature of serrated pathways is mutations in the *KRAS* or *BRAF* genes, leading to the hyperactivation of the MAPK kinase signaling pathway [24].

Another subset of sporadic CRC cases develops through different molecular pathways:

- Microsatellite Instability (MSI): Microsatellites are DNA sequences consisting of 1 to 6 base pair repeats distributed throughout the human genome, representing approximately 3% of the human genome and highly susceptible to mutations. The determination of their status is commonly used for tumor diagnosis and classification, as well as predicting and assessing treatment response [25]. MSI is a molecular alteration involving high mutability and affecting genes related to DNA mismatch repair (MMR), subdivided into high (MSI-H), low (MSI-L), or stable (MSI-S). MSI-H is observed in approximately 15–20% of CRC cases and is attributed to the hypermethylation of the promoters of the *hMSH2* (human homolog of the DNA mismatch repair gene 2) and *hMLH1* (human homolog of the DNA mismatch repair MutL gene) genes and germline mutations in DNA mismatch repair (MMR) genes [26]. MSI-H is commonly associated with Lynch syndrome, an inherited condition with a high risk of developing CRC [27,28]. Although MSI-H status does not show a benefit with adjuvant treatment with 5-fluorouracil in stage II disease, it is a positive prognostic biomarker in early stages of CRC and in patients with advanced or metastatic disease treated with immunotherapy [29].
- Chromosomal Instability (CIN): This results in changes in the number and structure of chromosomes and is the most common pathogenic pathway in CRC, contributing to approximately 84% of sporadic cases [30]. Most tumors originating in this pathway are primarily due to mutations in DNA repair genes, activation of oncogenes such as *PIK3CA* (phosphatidylinositol-4,5-bisphosphate 3-kinase catalytic subunit alpha) or *K-RAS* (Kirsten rat sarcoma virus), or inactivation of tumor suppressor genes such as *TP53* and *APC*. Mutations in the APC gene are characteristic of sporadic tumors and are present in over 80% of CRC cases, promoting initial clonal expansion and tumoral progression by activating the Wnt signaling pathway [31]. This pathway controls the proliferation, differentiation, and renewal of intestinal stem cells, leading to the formation of dysplastic crypts that can progress to adenomas [20]. Chromosomal instability can give rise to the Vogelstein model of adenoma–carcinoma–metastasis in 70–90% of CRC cases, characterized by mutations in APC, TP53, and DCC (deleted in CRC), resulting in the inhibition of apoptosis, increased cell proliferation, and reduced cell adhesion [32]. Additionally, approximately 10% of colorectal tumors evolve through morphological changes in a pathway known as serrated neoplasia [33].
- CpG Island Methylator Phenotype (CIMP): Involves hypermethylation of cytosine-guanine base pair repeats connected by phosphate (CpG sites or CpG islands) in gene promoter regions and has been associated with genomic imprinting, X chromosome inactivation, gene silencing, and carcinogenesis, especially when affecting tumor suppressor genes [34]. It is thought that CRC tumors with CIMP promoter methylation characteristics originate through the serrated neoplasia pathway and show markedly different histology compared to tumors derived from the traditional adenoma–carcinoma pathway [33,35,36,37].

In 2015, due to the complexity and variability of CRC, an international consortium, the Centers for Medicare and Medicaid Services (CMS), proposed a significant advancement in the molecular classification of this disease. This new classification system was based on information collected from 4000 CRC patients and considered not only molecular and genetic characteristics but also clinical and prognostic data of the disease [38]. As a result, four consensus molecular subtypes (CMS) were established. CMS1, termed “Immune MSI”, is found in 14% of cases and is characterized by microsatellite instability, hypermethylation, and immune cell infiltration. CMS2, called “Canonical”, occurs in 37% of cases and is associated with the activation of the WNT and MYC signaling pathways. CMS3, called “Metabolic”, is present in 13% of cases and is characterized by epithelial and metabolic dysregulation, *KRAS* gene mutations, and a combination of microsatellite instability and a CpG island methylator phenotype. Finally, CMS4, known as “Mesenchymal”, is found in 23% of cases and is associated with the activation of the TGF-β signaling pathway, stromal infiltration, and angiogenesis [39]. This molecular classification system is essential for better understanding CRC and guiding treatment decisions based on the specific characteristics of each subtype.

In this regard, the application of proteomics in the study of key molecular pathways, such as WNT, MAPK, TGFβ, and p53, has expanded our understanding of the mechanisms driving the initiation and progression of CRC [40,41,42]. The identification of specific proteins involved in these pathways has led to the development of more targeted therapies, offering new treatment options for patients. Additionally, the molecular classification of CMS, based on proteomic data, has improved patient stratification, allowing for a more precise and personalized approach to treatment [39,43].

## 3. Search and Validation of Protein Biomarkers in CRC

### 3.1. Diagnostic Biomarker

A diagnostic biomarker is a biological characteristic that indicates the presence of a disease or condition [44]. In the case of CRC, early diagnosis is key to reducing mortality [45], as a 5-year survival rate of approximately 90% is observed when detected at early stages, decreasing to around 14% at advanced stages [17].

Despite advances in diagnostic strategies, including imaging tests, colonoscopy, or fecal occult blood tests, there are associated barriers such as a lack of public participation in screenings and the discomfort associated with invasive diagnostic methods [46]. For this reason, non-invasive approaches, such as fecal immunochemical tests (FITs) and fecal DNA tests, have been explored, but their effectiveness still depends on a confirmation through colonoscopy [47]. This highlights the urgent need to identify early, specific, and sensitive biomarkers to enhance CRC screening strategies, where proteomic studies play a crucial role (Table 2).

Two-dimensional gel electrophoresis and mass spectrometry have been used to analyze CRC tissue samples, revealing the overexpression of proteins such as ACTBL2 and DPEP1 [48,49]. Additionally, the use of formalin-fixed paraffin-embedded (FFPE) tissues has expanded access to larger cohorts [68]. In this regard, mass spectrometry-based proteomics combined with machine learning analysis of FFPE tissues has been able to distinguish groups of proteins capable of predicting the future appearance of high-grade adenomas or CRC development [50].

On the other hand, the trend of searching for biomarkers through non-invasive approaches has emphasized the role of blood samples due to their accessibility and low risk. Different proteomic studies have identified panels of four to five proteins that show very good performance in early disease detection [51,52,53]. Similarly, Harlid and colleagues have identified that fibroblast growth factor 21 (FGF-21) and pancreatic prohormone (PPY) are associated with the risk of colon and rectal cancers, respectively, in plasma samples from asymptomatic patients and in a pre-diagnostic setting [54]. The most relevant aspect of this study is the authors’ emphasis on adding protein markers to basic CRC risk prediction models to increase their performance, since small protein biomarkers or panels alone may not be sufficient for effective precision detection.

In line with the search for non-invasive approaches, promising protein biomarkers in urine have also been identified, allowing for reliable detection and diagnosis of CRC, either alone or in combination with FIT [55]. Similarly, metastatic signatures that serve to stratify the risk have been identified, as they can predict over 50% of metastatic patients with a negative serum carcinoembryonic antigen (CEA) [55]. Likewise, two transmembrane proteins, CD147 and A33, have been identified in extracellular vesicles derived from the feces of CRC patients, which are inherently associated with the disease and could serve as protein biomarkers for non-invasive large-scale CRC detection [56].

Despite these advances, none of the identified protein biomarkers has reached clinical practice, possibly due to the difficulty of validation in large cohorts and comparison with current detection methods. Another common reason why biomarkers fail to achieve clinical use is that, unless confusing comorbidities are included in research studies, it is easy for researchers to mistakenly identify general markers of disease as specific markers for the cancer in question, especially when conducting simple case–control studies. This is true both at the metabolomic level [69] and at the proteomic level [70]. However, proteomic research in this field remains essential to fill the gap in CRC detection with reliable biomarkers and improve the early detection of this disease [71].

### 3.2. Predictive Biomarker

Predictive biomarkers are essential for personalizing and improving CRC treatment, especially with the increasing therapeutic options [72], and proteomics emerges as a valuable tool for their identification (Table 2).

In this regard, chemotherapy resistance poses a significant challenge in CRC treatments. Wang and colleagues compared proteomic, genomic, and transcriptomic profiles in CRC cells and tumors, finding that proteomic data have better potential to predict sensitivity to various drugs compared to genomic or transcriptomic data [73]. Others, such as Guo and his team, have investigated resistance, especially to oxaliplatin, a frontline treatment for metastatic CRC, identifying an overexpression of the PCBP1 protein in samples from resistant tumors [74]. The response to bevacizumab, a vascular endothelial growth factor inhibitor, has also been evaluated, where three proteins (APOE, AGT, and DBP) were identified, and their expression was correlated with better survival outcomes in patients treated with a combination of chemotherapy and bevacizumab [57]. Furthermore, the evaluation of the response to EGFR-targeted therapies, such as cetuximab, revealed that the plasma level of phosphorylated EGFR (pEGFR) was associated with therapy sensitivity [75].

Proteomic studies have also identified, through three protein folding stability profiling techniques, 10 proteins related to cancer chemoresistance, of which 2 have been validated in vitro, fatty acid synthase, and elongation factor 2 as pharmacological targets with biological functions that can be modulated to improve the efficacy of CRC chemotherapy [58].

In the context of neoadjuvant chemoradiation for rectal cancer, predicting the response is crucial. Proteomic studies have identified protein signatures in tumor biopsies that correlated with complete or non-responsive responses to therapy, providing valuable information for treatment planning [59].

In the field of immunotherapy, the antitumor immune response is under investigation. Tumors with MSI-H and mismatch repair deficiency respond better to immunotherapy. However, not all patients with MSI-H tumors respond, emphasizing the need for more specific biomarkers [29,76]. An immunoproteomic study using mass spectrometry identified potential immunotherapeutic targets. Yang and colleagues found a differential expression of proteins such as PSMA1, LAP3, ANXA3, and Maspin in CRC patients, suggesting an immunogenic proteomic profile associated with cancer [60,61].

Additionally, Redondo et al. demonstrated that an increase in clusterin protein expression is implicated in malignant progression, so its expression can help identify patients with more aggressive tumors who may benefit from targeted therapy [77].

Other studies, such as that of Yu et al., used magnetic beads and mass spectrometry to analyze sera from CRC patients, identifying the protein STK4 as a potential predictive marker for distant metastasis [62]. In this line of research, the positive regulation of proteins such as MRC1 and S100A9 in the serum of CRC patients has also been revealed, highlighting the diversity of potential biomarkers [63].

Despite advances in identifying predictive biomarkers, translating them into clinical practice faces the same challenges as diagnostic biomarkers. Ongoing research in this field is crucial to improve treatment response and move towards more personalized therapies for CRC patients.

### 3.3. Prognostic Biomarker

Similarly, prognostic biomarkers play a crucial role in managing CRC, providing information about overall outcomes regardless of therapy [78]. Although CEA remains the most widely used biomarker, its specificity is limited [79]. Other parameters, such as MSI and BRAF mutation, have been explored, but additional biomarkers are urgently needed to improve CRC treatment and monitoring [80].

The occurrence of metastases, especially in the liver, is an unfavorable prognostic factor in CRC [81]. Proteomic studies using techniques like SELDI and iTRAQ have identified specific proteins, such as HLAB, 14-3-3β, ADAMTS2, LTBP3, NME2, and JAG2, related to tumor progression and metastasis [82]. Additionally, collagen proteins, such as collagen type XII, have been shown to be promising candidates in the metastatic context [64]. In the detection of hepatic metastases, collagen peptides in urine and the measurement of the PGE-M metabolite are presented as promising and non-invasive approaches [65,83]. Studies have shown that these methods can be correlated with the risk of CRC [84,85].

To predict nodal status, protein biomarkers have been investigated. HSP47 and ezrin have proven to be relevant in identifying metastasis in lymph nodes [66,86], which could improve the guidance of chemotherapy and the extent of surgery.

Detecting postoperative recurrence is another significant challenge, and some studies using a reverse-phase protein array have identified eight proteins, including collagen VI, inositol polyphosphate-4-phosphatase, and Maspin, as significant prognostic factors for tumor recurrence [67]. Maspin has also been highlighted as an early recurrence marker in stage IV CRC [87].

Although several promising biomarkers have been identified (Table 2), CEA remains the only established prognostic biomarker in clinical practice. The search, validation, and clinical application of new biomarkers are essential to address current limitations in predicting nodal status, distant metastases, and postoperative recurrence in CRC.

## 4. Relevance of Samples in Proteomics and CRC

During the last two decades, global proteomic studies have witnessed a significant surge in protein identification, especially in serum and plasma, facilitated by liquid chromatography coupled with mass spectrometry (LC-MS) [88]. Although progress has been made in identifying blood biomarkers, their clinical validation has been limited. Technical and physiological complexities, such as the high complexity of blood samples and the predominance of certain proteins, have hindered the detection of less abundant biomarkers [89]. Despite the availability of advanced technologies, the discovery of new blood biomarkers has had limited success, and the gap between discovery and clinical utility remains a challenge [90]. As a result, some researchers have opted to focus on identifying tissue-level biomarkers before searching for them in the blood. In this context, formalin-fixed paraffin-embedded (FFPE) tissues have gained popularity as a viable alternative.

Although FFPE tissues were initially considered challenging for proteomic analysis, recent research has demonstrated the opposite. Between 2005 and 2007, studies revealed that it was possible to identify hundreds of proteins in FFPE tissues using mass spectrometry (MS) [91,92]. Long-term stability, widespread availability, and lower storage costs have contributed to the growing acceptance of FFPE tissues in proteomic analysis [93]. Protein extraction from FFPE tissues for proteomic analysis involves the use of buffers, detergents, heat, and, in some cases, pressure [94]. These methods have effectively been shown to reverse formalin cross-links, enabling protein identification. Pressure has demonstrated significant improvements in protein extraction from FFPE tissues, enhancing efficiency and the quantity of extracted proteins [95]. Despite advances, challenges persist, such as the need for standardization in sample preparation and concerns about the complete reversal of formalin cross-links. Although kits and technologies have been developed to simplify protein extraction from FFPE tissues, further research is still required to optimize and standardize these processes [96,97].

The proteomic analysis of FFPE tissues has gained popularity in biomedical research, marking a significant shift in the understanding and application of these samples. As the viability of FFPE tissues for proteomic analysis became recognized, there was an increase in the scale of biomarker studies [98]. The number of quantifiable proteins in FFPE tissues went from hundreds to thousands, and post-translational modifications such as phosphorylation and glycosylation were explored [99]. Until December 2022, 432 articles related to “FFPE” and “mass spectrometry” were registered in PubMed, compared to 52 at the end of 2010 [100].

The SP3-CTP method (single-pot solid-phase-enhanced clinical tissue proteomics sample preparation) emerged as a high-throughput approach to quantitatively compare proteins in hundreds of FFPE tissues. This method involves tissue deparaffinization, followed by enzymatic lysis, protein reduction, and alkylation. Proteins bind to magnetic beads, undergo washing, and undergo enzymatic digestion. The resulting peptides are quantified using tandem mass tag (TMT) labels, enabling comparison between samples [101]. A study applied SP3-CTP to profile the proteomes of 300 FFPE breast tumors and 38 normal tissues. The goal was to improve the classification of tumors according to PAM50 subtypes. The analysis identified four groups with distinctive characteristics, such as specific metabolisms and immune responses. For triple-negative breast cancer (TNBC), four subgroups with unique proteomic profiles were identified, related to immune response, extracellular matrix, lipid metabolism, and DNA replication [102]. The correlation between proteomic groups and PAM50 classifications suggests potential clinical applications. It was highlighted that tumors with an abundance of immune proteins exhibited higher survival rates, emphasizing the importance of these biomarkers in therapeutic guidance [101].

Immunohistochemical analysis (IHC), although the standard for tissue classification, has limitations in terms of subjectivity and a low resolution [103]. In contrast, the Liquid Tissue-SRM (selected reaction monitoring mass spectrometry) method offers advantages by not requiring antibodies and allowing objective quantification of biomarkers, as demonstrated in the measurement of MET and Her2 in FFPE tumors [104,105,106]. Despite advances, challenges persist, such as the lack of standardization in FFPE tissue preparation, affecting the representativeness of proteomes. Variability in fixation time and the lack of standardized protocols are key concerns. Additionally, the precise identification of peptides modified through formalin fixation remains a technical challenge [98].

## 5. Conclusions

Research related to colorectal cancer has experienced significant advances in recent years owing to the application of proteomics, a discipline that allows for the systematic study of proteins and their interactions within biological systems. Progress in this field has transformed the understanding of the complex molecular alterations associated with CRC, providing valuable insights for diagnosis, prognosis, and the development of personalized treatments.

In this regard, proteomics has enabled a more detailed characterization of molecular alterations in CRC, creating proteomic profiles associated with different stages of the disease. Likewise, potential biomarkers have been identified, opening new avenues for early diagnosis and patient stratification, crucial for improving survival rates. Moreover, the ability to analyze biological samples from tumor tissue or bodily fluids using these techniques has allowed for a better differentiation of CRC subtypes and identification of the influence of various factors, providing valuable information for clinical decision-making.

However, the contribution of proteomics goes beyond diagnosis, extending to prognosis and predicting treatment response, as specific biomarkers have been identified, enabling more personalized treatment strategies, minimizing exposure to ineffective therapies, and, thus, supporting the transition to precision medicine.

Despite possible achievements, CRC research still faces significant challenges such as the complexity of biological samples, method standardization, and the management of large datasets, requiring multidisciplinary collaboration and more innovative approaches. Additionally, the clinical validation of biomarkers and therapeutic targets identified through proteomics is necessary for their successful implementation in clinical practice. Therefore, it is essential to emphasize the importance of continuing proteomic research in CRC, as the constant evolution of proteomic technologies, combined with a deeper understanding of the molecular complexities of CRC, will open new opportunities for innovation and the development of more effective strategies.

## Figures and Tables

**Table 1:** Characteristics of High-Complexity Proteomic Technologies.

	Basis Principles	Advantages	Disadvantages
Mass spectrometry (MS) [3]	Targeted samples, digestion, peptide ionization, and tandem MS scans	De novo process suitable for exploratory research	Low throughput, complex depletion process, limitations for analyzing protein PTMs
Reverse-phase protein arrays (RPPA) [4]	Samples immobilized on solid substrates and antibody-detected targets	Large scale analysis of samples	Relatively long turnaround time
Antibody/antigen arrays [5]	Protein-targeted immobilized samples on solid substrates in antibody/antigen-captured samples	Flexible experimental design and PTM profiling	Inter-assay reproducibility and quantification limit, inter-assay variation and sample labeling
Proximity extension assays (PEA) [6]	Sandwich ELISA with labeled complementary DNA oligos	Small sample for large dynamic traits	Requires qPCR/NGS for reading
Aptamer-based assays [7]	Short single-stranded DNA or RNA folded into tertiary structures with ability to bind to targets with high affinity and specificity	High complexity	Reliance on DNA microarrays for readout

**Table 2:** Potential biomarkers identified, sample type, and proteomic technology used.

Utility	Protein	Sample	Proteomic Technologies	References
Diagnostic	ACTBL2 and DPEP1	Fresh tissues	Two-dimensional gel electrophoresis and mass spectrometry	[48,49]
	C1QBP, ERGIC1, and ORMDL1	FFPE tissues	Mass spectrometry-based proteomics combined with machine learning analysis	[50]
	Leucine-rich alpha-2 glycoprotein 1, epidermal growth factor receptor, inter-alpha-trypsin inhibitor heavy-chain family member 4, hemopexin, and superoxide dismutase 3	Serum	Targeted liquid chromatography-tandem mass spectrometry	[51]
	Mannan binding lectin serine protease 1, osteopontin, serum paraoxonase lactonase 3, and transferring receptor protein 1	Plasma	Liquid chromatography/multiple reaction monitoring-mass spectrometry (LC/MRM-MS) and PEA	[52]
	CD79B, DDR1, EFNA4, FLRT2, LTA4H, and NCR1	Plasma	PEA assay	[53]
	FGF-21 and PPY	Plasma	PEA assays	[54]
	COROC1C, RAD23B, and ARPC3	Urine	LC/MS-MS	[55]
	CD147 and A33	Extracellular vesicles derived from the feces	Western blot	[56]
	APOE, AGT, and DBP	Serum	LC/MS-MS	[57]
	Fatty acid synthase and elongation factor 2		Protein folding stability profiling techniques	[58]
	IFIT1, FASTKD2, PIP4K2B, ARID1B, and SLC25A33	FFPE tissue	MS	[59]
	PSMA1, LAP3, ANXA3, and Maspin	Tissue	MS	[60,61]
	STK4	Tissue	Magnetic beads and mass spectrometry	[62]
	MRC1 and S100A9	Serum	LC/MS-MS	[63]
Prognostic	HLAB, 14-2-3β, ADAMTS2, LTBP3, NME2, and JAG2	Tissue	SELDI and iTRAQ	[64]
	Collagen type XII	Urine	LC/MS-MS	[65]
	HSP47	Tissue	iTRAQ	[66]
	Collagen VI, inositol polyphosphate-4-phosphatase, and Maspin	Tissue	Reverse-phase protein array	[67]
